# Recent Advances in Self-Exciting Photodynamic Therapy

**DOI:** 10.3389/fbioe.2020.594491

**Published:** 2020-10-20

**Authors:** Nicholas Thomas Blum, Yifan Zhang, Junle Qu, Jing Lin, Peng Huang

**Affiliations:** ^1^Marshall Laboratory of Biomedical Engineering, Laboratory of Evolutionary Theranostics (LET), International Cancer Center, School of Biomedical Engineering, Shenzhen University Health Science Center, Shenzhen, China; ^2^Key Laboratory of Optoelectronic Devices and Systems of Ministry of Education and Guangdong Province, College of Optoelectronic Engineering, Shenzhen University, Shenzhen, China

**Keywords:** nanoplatform, photodynamic therapy, ROS, chemiluminescence, bioluminescence, Cerenkov, cancer

## Abstract

Photodynamic therapy (PDT) is already (Food and Drug Administration) FDA approved and used in the clinic for oncological treatment of pancreatic, lung, esophagus, bile duct, and of course several cancers of skin. It is an important tool in the oncological array of treatments, but for it exist several shortcomings, the most prominent of which is the shallow depth penetration of light within tissues. One-way researchers have attempted to circumvent this is through the creation of self-exciting “auto-PDT” nanoplatforms, which do not require the presence of an external light source to drive the PDT process. Instead, these platforms are driven either through oxidative chemical excitation in the form of chemiluminescence or radiological excitation from beta-emitting isotopes in the form of Cherenkov luminescence. In both, electronic excitations are generated and then transferred to the photosensitizer (PS) *via* Resonance Energy Transfer (RET) or Cherenkov Radiation Energy Transfer (CRET). Self-driven PDT has many components, so in this review, using contemporary examples from literature, we will breakdown the important concepts, strategies, and rationale behind the design of these self-propagating PDT nanoplatforms and critically review the aspects which make them successful and different from conventional PDT. Particular focus is given to the mechanisms of excitation and the different methods of transfer of excited electronic energy to the photosensitizer as well as the resulting therapeutic effect. The papers reviewed herein will be critiqued for their apparent therapeutic efficiency, and a basic rationale will be developed for what qualities are necessary to constitute an “effective” auto-PDT platform. This review will take a biomaterial engineering approach to the review of the auto-PDT platforms and the intended audience includes researchers in the field looking for a new perspective on PDT nanoplatforms as well as other material scientists and engineers looking to understand the mechanisms and relations between different parts of the complex “auto-PDT” system.

## Introduction

Photodynamic therapy (PDT) has been approved by the Food Drug Administration (FDA) to treat a variety of tumors and malignancies in the clinic (Da̧browski and Arnaut, 2015; van Straten et al., 2017). While effective, the primary limitation is the penetration of light within human tissue (Bashkatov et al., 2005, 2011) restricting applications essentially to areas where light can be directly applied like skin, lungs, and partially resected tumors (Bargo and Jacques, 2001; Huang, 2005; Cohen and Lee, 2016; Naidoo et al., 2018). PDT works by illuminating a molecule belonging to a class of compounds known as photosensitizers (PSs). Electrons excited by the incoming radiation will jump to the higher energy orbital in the PS, after which two things may happen. One is the electron immediately relaxes back to the ground state (time scale ∼10^–10^ s) (Laor et al., 1973; DeRosa, 2002; Zhao et al., 2013b); this is the unproductive pathway for PDT. The other pathway is to undergo intersystem crossing, a spin forbidden electronic orbital transition (∼10^–8^ s), and then react with, most typically, an oxygen molecule generating singlet oxygen (Menzel and Thiel, 1998; DeRosa, 2002; Zhao et al., 2013b). Singlet oxygen is a highly reactive oxygen species (ROS) which oxidizes biological compounds, causing cell damage and stress (Dougherty, 1987; Clennan, 2000). This is the productive pathway where PDT is concerned; PSs are different from typical fluorescent compounds because their fluorescent quantum efficiency is quite low; they are designed or chosen to have high rates of intersystem crossing, making ROS generation the preferred pathway (Zhou et al., 2016). ROS induced cell damage can be localized with *via* specific light illumination, as this generation of ROS’s does not occur outside of light illumination, sparing non-illuminated, healthy cells. In this way, PDT can effectively target cancerous cells and has led to effective treatments where light can easily be applied.

Of course, this entire process begins with excitation from an externally applied light source, however, one of the main disadvantages is the limited penetration depth of light in animal tissue (Bashkatov et al., 2005, 2011). Particularly as in the case for inoperable cancers, or those sitting deep within organs or other tissues, which are generally unable to be treated by conventional PDT. Besides the limited penetration depth of light, there is also the issue of limited cellular uptake of PS (Windahl et al., 1993; Miller et al., 1994; Costanzo et al., 2016), which may reduce therapeutic efficacy. There are several recent examples of clinically relevant efficacy for PDT treatment of cancers (Kawczyk-Krupka et al., 2015; Hauge et al., 2016; Almerie et al., 2017; Rizzo et al., 2018; DeWitt et al., 2019; Fisher et al., 2019; Mahmoudi et al., 2019), so it would be beneficial to bring these same therapeutic effects to deep-seated tumors. These therapeutic effects are numerous and there are a few aspects which elevate PDT as compared to other competing treatments (e.g., chemotherapy, radiation therapy). For example, PDT mediated therapies do not generally suffer from whole body toxicity for therapeutic effect, because the applied light is site-specific and the PSs are only activated in the illuminated area. Moreover, healthy cells have been reported to be able to tolerate increased ROS stress better than cancer cells (Trachootham et al., 2006, 2009; Cairns et al., 2011). PDT has also been shown to elicit very strong immune responses which has been shown to decrease resultant tumor size or in some cases shrink the tumor mass below the initial size for robust anti-tumor immunity when combined with checkpoint inhibitors (Duan et al., 2016; Xu et al., 2017; Yang G. et al., 2018; Liu et al., 2019).

**SCHEME 1:**
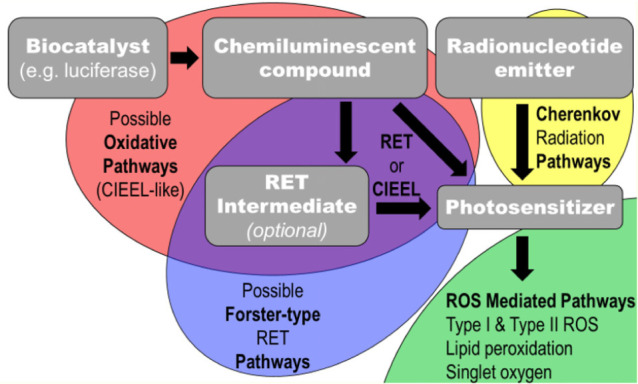
An abstracted flow chart for self-exciting PDT pathways and significant components. In this this scheme, gray boxes indicate important physical components to self-exciting PDT nanoplatforms and the semi-transparent ellipses indicate possible pathways of possible energy transfers; the black arrows indicate transfer of energy to or excitation of the compound being pointed to.

Thus, biomedical and materials scientists began looking for ways to excite PSs using chemical or radiological methods. Many of these methods can be grouped into a class of therapies known as “Auto-PDT” (APDT), where the PDT is initiated from compounds co-administered or co-loaded with the PSs. The advantage of this type of excitation lies in that the excitation of the PS is then independent of the applied light source, eliminating the major shortcoming of PDT-based treatment (Kotagiri et al., 2015; Fan et al., 2016). APDT is defined in this review as the self-driven excitation of the PS from other compounds or materials injected into the body. The electronic excitation, either chemical or radiological in origin, is transferred to the PS which can then generate ROSs in the absence of any externally (i.e., from outside of the body) applied radiation or trigger. A scheme showing the important components and possible pathways within an APDT system is depicted in Scheme 1.

This review uses a materials standpoint to focus on the excitation schemes and compositions for the construction of a self-exciting PDT system, with emphasis given to the corresponding mechanisms of PDT. First, the mechanisms of excitation of the PS are discussed and separated into four sections: intermolecular chemically induced electron exchange excitation (CIEEL-like), Resonance Energy Transfer (RET), Two-Stage RET, and Cherenkov Radiation Energy Transfer (CRET). CRET in this review will always refer to CRET and never Chemiluminescent RET. Then, the types of nanoparticle platforms are categorized by their essential function and coupling of functional components. This review provides a critical view on the terminology, claims, and proposed mechanisms in the included papers; in turn, a basic design strategy is prescribed for the development of auto-PDT nanoparticle systems, while identifying challenges and themes in their synthesis and application. These analyses are necessary for accurate portrayal of the materials science in an emerging field.

## Method of Electronic Excitation

In most cases, APDT therapies are restricted in the use of an excitation donor far more so than PSs, as the library of known chemiluminescent compounds, and materials that emit beta radiation, are comparatively small. As a result, self-exciting PDT materials and strategies usually concern themselves primarily with methods to incorporate one of the few available excitation methods, then choose a suitable PS. Thus, in this review, the focus will be on the method of excitation, and the choice of PS will be largely ignored except where directly relevant. Method of excitation, PS choice, and composition of the of key papers referenced herein can be found in Table 1.

**TABLE 1:** A summary of the key papers for nanoplatforms for self-exciting photodynamic therapy with associated key aspects.

Author	Nanoparticle type	Excitation type	Transfer method	Exciting component	Photosensitizer	Components	Year
Lin et al.	MOF	Bioluminescence	RET	D-fluorescein	TCPP	CL, PS	2019
Yang et al.	PLGA	Bioluminescence	RET	Luciferin	RB	Bio. Cat., PS	2018
Hsu et al.	QDs	Bioluminescence	RET w/Int.	Coelenterazine	m-THPC	RET Int., Bio. Cat.	2013
Kim et al.	QDs	Bioluminescence	RET w/Int.	Coelenterazine	Ce6	RET Int., Bio. Cat.	2015
Yang et al.	CDs	Bioluminescence	RET w/Int.	Luciferin	PPIX	RET Int., PS	2018
Zhao et al.	Microcapsules	Bioluminescence	RET	Luciferin	RB + Hypocrellin B	CL, Bio. Cat., PS	2013
Yu et al.	HMSN	Chemiluminescence	CIEEL + RET	CPPO	Ce6	CL, PS	2018
Xu et al.	Self-assembled NP	Chemiluminescence	RET	Luminol	Ce6	CL, PS	2019
Fang et al.	MOF	Chemiluminescence	RET	Luminol	TCPP	CL, PS	2019
An et al.	Self-assembled NP	Chemiluminescence	RET	Luminol	Ce6	CL, PS	2020
Al-Ani et al.	Protein NP	Chemiluminescence	RET	Coelenterazine	ZnPP	Bio. Cat., PS	2019
Jiang et al.	Protein/polymer NP	Chemiluminescence	RET	Luminol	MEH-PPV	PS	2019
Berwin Singh et al.	Polymer Micelle	Chemiluminescence	CIEEL	Peroxalate Polymer	PPIX	CL, PS	2017
Yang et al.	Functionalized CDs	Chemiluminescence	RET w/Int.	Luminol	Ce6	RET Int., PS	2019
Wu et al.	Polymer NPs	Chemiluminescence	CIEEL	CPPO	TPP	CL, PS, RET Int.	2019
Zhang et al.	Polymer NPs	Chemiluminescence	RET	Luminol	m-THPC	RET Int., PS	2014
Kotagiri et al.	TiO2 NP	Cherenkov Radiation	CRET	FDG	TiO2	BNE, PS	2015
Kamkaew et al.	HMSN	Cherenkov Radiation	CRET	^89^Zr	Ce6	BNE, PS	2016
Ni et al.	MNP	Cherenkov Radiation	CRET	^89^Zr	TCPP	BNE, PS	2018

*Metal organic framework, MOF; poly(D,L-lactide-co-glycolide), PLGA; quantum dots, QD; carbon dots, CD; hollow mesoporous silica nanoparticle, HMSN; nanoparticle, NP; magnetic nanoparticle, MNP; resonance energy transfer, RET; RET with Intermediates, RET w/Int.; chemically induced electron exchange luminescence type mechanism, CIEEL; bis(2,3,5-trichloro-6-((pentyloxy)carbonyl)phenyl) oxalate, CPPO; 2′-deoxy-2′-(^18^F)fluoro-D-glucose, FDG; tetra (4-carboxyphenyl) porphyrin, TCPP; Rose Bengal, RB; meta-tetra(hydroxyphenyl)chlorin, m-THPC; Chlorin e6, Ce6; Zinc (II)-protoporphyrin IX, ZnPP; protoporphyrin IX, PPIX; poly[2-methoxy-5-(2-ethylhexyloxy)-1,4-phenylenevinylene], MEH-PPV; tetraphenylporphyrin, TPP; Chemiluminescent compound, CL; Bioluminescence catalyst, Bio. Cat.; photosensitizer, PS; Beta nucleotide emitter, BNE.*

### Intermolecular Chemically Induced Electronic Excitation

The most fundamental, but perhaps the least commonly applied, method to excite the PS is direct excitation of the PS *via* molecular chemically initiated electron excitation. Chemically initiated electron exchange luminescence (CIEEL) was first posited by Schuster (Koo and Schuster, 1978; Schuster, 1979), but many mechanistic studies have since shown the basis of his theory flawed, particularly in the way of unexplained low quantum efficiencies for certain CL reactions (Catalani and Wilson, 1989; Isobe et al., 2005; Almeida de Oliveira et al., 2012; Yue et al., 2012). Many revised and a few alternative mechanisms (e.g., ICIC) (Pinto da Silva and Esteves da Silva, 2013a, b) now exist but no one theory is currently accepted to explain all facets of the chemiexcitation (Augusto et al., 2013). In this paper, we will use the term CIEEL to refer to the collection of revised mechanisms as a whole (CIEEL-like) and the apparent direct chemical excitation reported in some papers for certain CL and PS compounds. Due to the complexity of CIEEL-like excitation chemistry as compared to other methods, i.e., RET and CRET, relatively few papers have used this method for excitation of PS compounds. CIEEL-like excitation requires delicate tuning of oxidation potentials *via* organic syntheses (Shim et al., 1997; Zhen et al., 2016), whereas RET and, to a lesser extent, CRET merely require information about the emission and excitation spectra of the molecule undergoing chemical excitation and the PS, respectively, in order to be properly applied.

**FIGURE 1:**
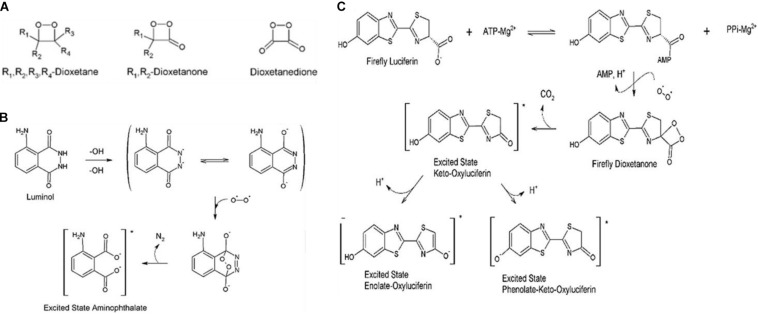
Mechanisms chemical and biochemical luminescence. **(A)** Generalized chemical formulae for possible dioxetanedione structure. **(B)** Luminescence mechanism for luminol. **(C)** Mechanistic pathways for firefly luciferin, catalyzed by firefly luciferase in the second reaction step, toward bioluminescence (Magalhães et al., 2016).

Since the CIEEL mechanism is covered in detail by many papers, an expanded discussion will not take place here (Schuster, 1979; Stevani et al., 2000; Orlova et al., 2003; Matsumoto, 2004; Ciscato et al., 2009). The key points of the above mechanisms, by which nearly all bioluminescent compounds as well as many chemiluminescent compounds produce light, are the formation of a 1,2-dioxetanone ring (Figure 1A), the presence of an electron donator, and the creation of an excited electronic state within the electron donating compound or within the dioxane containing compound itself, corresponding to intermolecular or intramolecular CIEEL-like CL, respectively, after the dioxetane ring decomposes. The excited electronic state can then relax to the ground state emitting an electron or transfer the excited energy *via* RET as will be seen later in this review. Mechanisms for the chemiluminescence of two of the most commonly utilized chemiluminescent compounds luminol and firefly luciferin can be seen in Figures 1B,C, respectively.

In a paper published by Mao et al. (2017), it can be seen how a chemiluminescent compound such as Bis[2,4,5-trichloro-6-(pentyloxycarbonyl)phenyl] oxalate (CPPO) can be used to directly excite a custom-made PS compound, named TBD, for APDT of a 4T1 xenograft cell cancer cell line in BALB/c mice.

**FIGURE 2:**
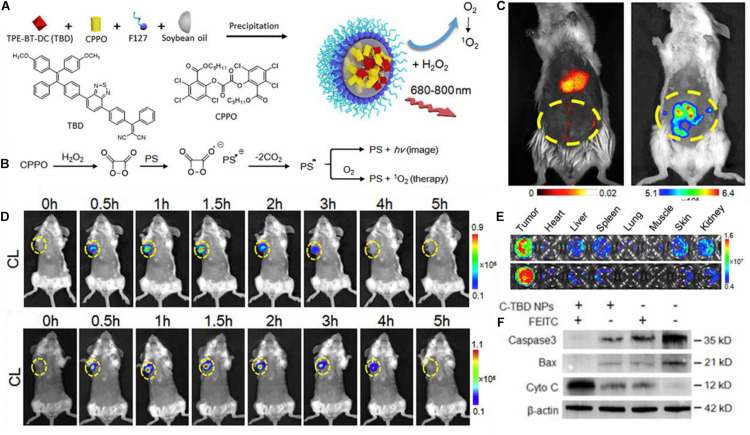
**(A)** A schematic showing the chemical structure of TBD and CPPO and their co-precipitation into a soybean oil droplet stabilized by F127 amphiphilic polymer. **(B)** The proposed mechanism by which CPPO can undergo CIEEL with the PS TBD for direct excitation of the PS for chemiluminescence or singlet oxygen generation. **(C)** Fluorescence imaging (left) vs. chemiluminescence imaging (right) of the TBD group imaging of intraperitoneal metastatic tumor bear mice after administration of C-TBD NPs; chemiluminescence occurs only in the intraperitoneal area, whereas fluorescence mainly occurs in the liver. **(D)** Chemiluminescence of mice with C-TBD NPs intravenously administered at 100 μL (1 mg/mL TBD) (upper) and after co-administration of anti-oxidant inhibiting agent, FEITC (5 μmol) (lower); persistence of chemiluminescent signals is extended for an additional hour. Also note the change in the chemiluminescent scale. **(E)** Chemiluminescent signals from lysates of major organs after adding C-TBD NPs directly to the well. **(F)** Western blot assay showing decreased Caspase3 and Bax, and increased cytochrome C, with C-TBD NPs and FEITC addition (Mao et al., 2017).

The nanoparticle was formed by co-precipitation of the TBD and CPPO in soybean oil droplets, stabilized by F127 polymer (Figure 2A). Figure 2B shows the theorized CIEEL mechanism for the excitation of the PS TBD where it acts as the electron donor, and, as the dioxetane ring decomposes, becomes excited. It can either then fluoresce or undergo intersystem crossing and generate singlet oxygen. Figure 2C shows an intraperitoneal cancer model with administration of C-TBD NPs where fluorescence imaging and chemiluminescent imaging show different biodistributions of the nanotheranostic agent. It is important to note that chemiluminescence generally occurs independently of PS excitation. For direct intermolecular CIEEL-like excitation of PSs, the chemiluminescence is the same as fluorescence in terms of the wavelength emitted, because there is only one compound capable of undergoing electronic excitation. The difference in chemiluminescence and fluorescence is whether or not an external light needs to be applied to observe fluorescence. In this case, and this terminology will be held consistent within this review, fluorescence shows the biodistribution of TBD (or another PS) when exposed to an external light source, and chemiluminescence is imaging in the dark (no external excitation). Thus, TBD is required to be present for both but only in the presence of CPPO and ROSs does it undergo luminescence in the dark.

Other images within the report show colocalization of the fluorescence and chemiluminescence in the tumor, and Figure 2C shows how the distribution of can appear significantly different. Preferential accumulation of the PS appears in the liver, but there is an obvious absence of chemiluminescence. It is also plausible that the nanotheranostic agent is not retaining its original structure; partial or complete breakdown of the theranostic could result in such images. The authors support their proposed mechanism of ROS induced therapy by adding a therapeutic adjuvant β-phenylethyl isothiocyanate (FEITC) which enhances H_2_O_2_ levels in cells (Figure 2D). The addition of FEITC increased the intensity and persistence of detectable chemiluminescent signal from the tumor. Moreover, lysates of various organs show that only tumor tissue generates significant H_2_O_2_ as determined by chemiluminescent imaging (Figure 2E), but this is subject to the caveat from part C where it is clear there is an unexplained absence of CPPO in the liver despite TBD presence; this should not occur if the nanoparticle formulation is intact. Further, the authors showed supportive results in terms of the proposed therapeutic mechanisms; they ran gels on the tumor cell cytosol which showed lowered levels of Caspase3 and Bax, with increased levels of cytosolic C (Figure 2D). The way these results are presented indicate several indicators of apoptotic activity, but the authors fail to propose any concerted mechanism correlating the observed phenomena.

There are scant other reports that claim to use CIEEL directly to excite the PS (Berwin Singh et al., 2017; Wu et al., 2019), and this is likely because adequate excitation of the PS involves careful tuning of the oxidation potentials of the chemiexcited donor and the recipient PS. However, this does not preclude the possibility that some reports have not correctly identified the precise pathway by which PSs are excited in an APDT system.

### Resonance Energy Transfer (RET) Excitation

In terms of popularity, the most common excitation methods for self-propagating PDT therapies in the absence of light are those supplied by chemiluminescence which is subsequently transferred to the PS *via* RET. RET will be used in the broadest sense of RET, including the subtype Forster resonance energy transfer. FRET cannot be used to described generally the excitation of PS by CL because many of the assumptions made by Forster break down in biological and nanoscale systems, both of which are relevant in the reviewed works. Bioluminescence resonance energy transfer (BRET) and chemiluminescent resonance energy transfer will both be referred to as resonance energy transfer (RET) since the mechanism of energy transfer is identical. Attempts will be made to keep in line with the terminology of the referenced papers and figures where appropriate, so BRET will sometimes be used interchangeably with RET.

An ideal chemiluminescent compound will emit light in the blue wavelengths, as the energy potential should sufficiently large to excite, *via* RET, most photosensitizing compounds, given that energy is always lost during a RET transfer (Jares-Erijman and Jovin, 2003). However, a blue light emission spectrum in the RET donor is not required, since the efficiency of RET is related to the overlap of the donor emission and acceptor absorption spectra (Haugland et al., 1969; Scholes, 2003). Regardless, many chemifluorescent compounds do emit in the blue or green wavelengths, which generally have high attenuation in biological tissues (Bashkatov et al., 2005), effectively ensuring that virtually all excitation of the PS ought not to come from the actual emission of the photon from the donor, but rather the RET process itself. This is distinct from CIEEL-like mechanisms, because CIEEL is a chemical oxidative mechanism by which a molecule can enter an excited state. RET PS excitation occurs when the chemiluminescent molecule is already in an excited state (such a state can arise through a CIEEL-like mechanism) but then transfers the energy to a PS *via* FRET.

Perhaps the most studied chemiluminescent compound is luminol, which was discovered in 1928 to react with blood to fluoresce more brightly as a result of the iron (and likely other substances) in blood which catalyzes the oxidation of luminol (Albreacht, 1928). Luminol was first used as a source for *in situ* PDT in Chen et al. (2012) where it was shown that by adding luminol to a cellular solution with FeSO_4_ and 5-aminolevulinic acid. Cancer cell viability dropped to 18% in flow cytometry experiments. FeSO_4_ served as the oxidative catalyst acting on luminol, with 5-aminolevulinic acid converted *via* cell metabolism into the photosensitizer protoporphyrin IX, which generated the ROSs necessary to induce cell toxicity (Chen et al., 2012). This work was closely followed by publication from Yuan et al. (2012) where luminol stimulated PDT was used an anti-cancer and anti-fungal agent for *in vitro* and *in vivo* experiments. Similar drops in cancer cell viabilities were observed in addition to a ∼60% decrease in tumor growth of a xenograft model.

Despite luminol being the most well-studied and well-known chemiluminescent compound, perhaps the most popular method for FRET induced excitation of PS is *via* the use of luciferases and luciferins, particularly that of firefly luciferase (fLuc) and its luciferin. It is important to note that by looking at the CIEEL-like mechanism for firefly luciferin excitation (Figure 1C), one may notice that the excited electronic energy does indeed come from the luciferin, and the luciferase merely catalyzes the oxidation. Nevertheless, many researchers and reports refer to luciferase as emitting the photon. In any case, the excited electronic state before photon emission exists on the order of nanoseconds, and the reaction absolutely does not proceed without the luciferase, so it is not functionally incorrect in terms of effect. We will use the term luciferase similarly in this review.

**FIGURE 3:**
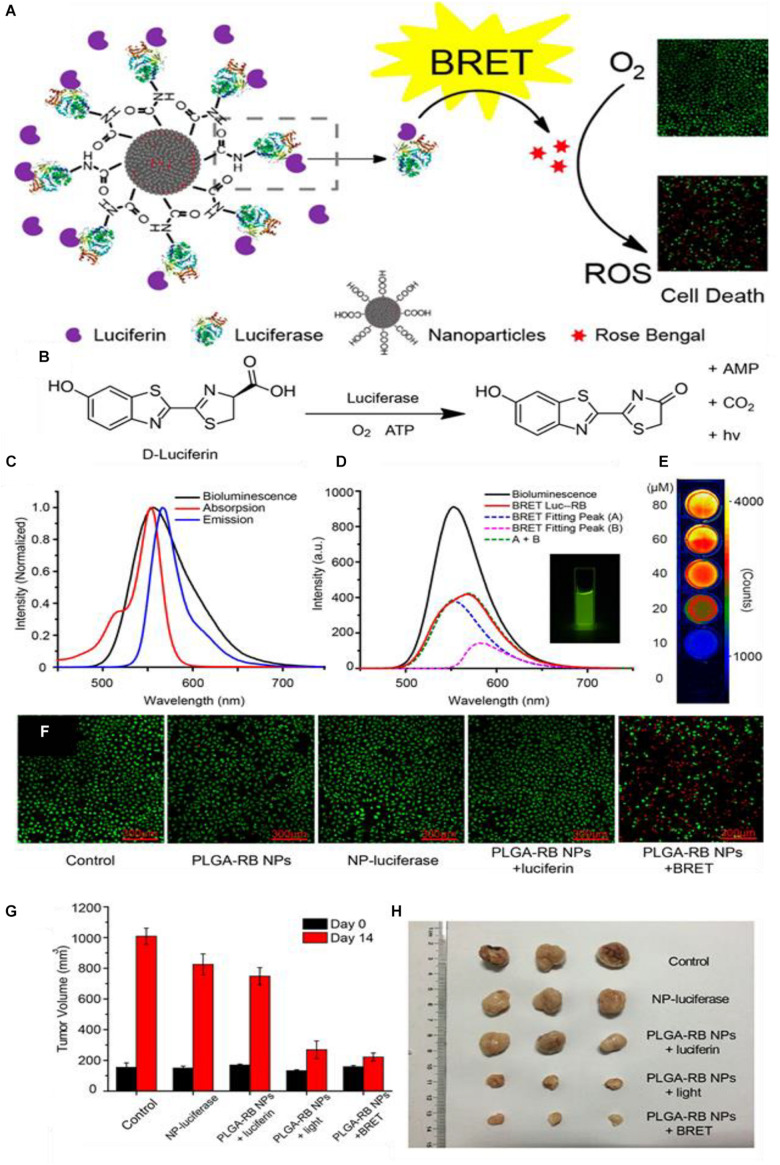
**(A)** Generalized scheme of the synthesis of the PLGA-RB nanoparticles: RB is first conjugated to PLGA then precipitated to form the nanoparticles, after which the luciferase is conjugated to the PLGA-RB surface. The luciferase can catalyze the oxidative luciferin electronic excitation which then transfers the energy *via* BRET to the RB. The RB reacts with oxygen generate ROSs for PDT. **(B)** The reaction scheme for the luciferin light emission which requires the luciferase, oxygen, and ATP. **(C)** Bioluminescent spectra of the luciferase-luciferin system, absorption, and emission of the PLGA-RB NPs. **(D)** Bioluminescent emission spectra of luciferase luciferin system, the emission spectra of the BRET Luc-RB system which is composed of fLuc mediated emission (Fitting peak A) plus fluorescent emission from RB (Fitting peak B); inset: emission of Luc-RB system. **(E)** Dose dependent emission based on varying luciferin concentrations. **(F)** Cell viability for different treatments measured by fluorescence 40 μg/mL PLGA-RB NPs with calcein-AM and propidium iodide stains; red fluorescence indicates dead cells. **(G)** End-point tumor size comparison of treatments and **(H)** photographs (citealpBR93).

In a report by Yang Y. et al. (2018), they show how Rose-Bengal (RB) can be functionalized to poly(lactic acid) (PLA), then condensed into a poly(lactic-coglycolide acid) (PLGA) nanoparticle (Figure 3A). The resulting nanocomplex is then functionalized with fLuc on the surface *via* N-ethyl-N-(3-(dimethylamino)propyl)carbodiimide (EDC) mediated conjugation; the luciferin is applied independently as a free molecule. In the presence of fLuc, chemi-excitation occurs of the luciferin (Figure 3B), and this is transferred *via* FRET to the RB PS. In the presence of luciferin but without RB, the fLuc spectrum as seen in the “bioluminescence” emission curve and inset in Figure 3C. With the addition of RB, and the associated occurrence of FRET, the intensity of the emissions is reduced overall. Bioluminescence of the fLuc complex still occurs (Fitting Peak A) but much of the energy is transferred via RET to the RB PS, which undergoes fluorescence in its PDT non-productive pathway (Fitting Peak B); both curves combine to make a new, lower intensity fluorescence (A + B, BRET Luc-RB) (Figure 3D). Additional electronic energy which would otherwise be emitted is lost during RET and when RB undergoes intersystem crossing to produce singlet oxygen. Figure 3E shows a light emission heatmap of wells with varying concentrations of luciferin. As expected, increasing luciferin concentration increases light emission, however, the paper does not explicitly specify the wavelengths used, an omission which is serious when considering RET systems. Figure 3F shows fluorescence images of stained live-dead cells; notably, there is a decrease in cell density in addition to generation of dead cells in the NP BRET treatment group.

When Wu and coworkers applied their PLGA-RD BRET formulation to an *in vivo* model, they found tumor growth suppression curves like those found when the PLGA-RD nanoparticles were irradiated with light and corresponding endpoint tumor volumes (Figures 3G,H). These results are promising as far as RET-driven PDT therapy is concerned; as they imply that while the intensity of catalyzed luminescence by fLuc and other luciferases are considerably lower in intensity than that of applied external light source, they can still be effective for PDT. There have been several studies which have attempted to quantify and compare the true PDT efficiencies of an external as compared to an internal tumor light source. Some authors have published conflicting results, notably with Dr. Gambhir and coworkers claiming that the bioluminescence produced by luciferins is too weak in luminescent intensity as compared to traditional PDT to initiate any significant toxicity (Theodossiou et al., 2003; Schipper et al., 2006; Magalhães et al., 2016; Shramova et al., 2018). Such uncertainty has not stopped the continued application of such systems in APDT; given the now numerous nanoparticle APDT papers showing efficacious treatment of cancer cells and tumors, it may be very tentatively said that RET in these formulations is sufficiently efficient to activate PS in an *in vivo* model. This may be due to inconsistencies in the ways these comparisons are made, or indeed, inclusion of APDT systems in a nanoparticle formulation allows for enhanced toxicity over free APDT systems.

### Two-Stage Photosensitizer Excitation/Excitation by RET Intermediary

One of the ways the PDT efficacy with RET can be improved is *via* addition of an intermediary. This intermediary usually has an excitation band gap that is between that of the CL compound and that of the PS acceptor. If the choice of a PS and CL compound do not have overlapping absorption and emission spectra, respectively, or whose spectra overlap poorly, then researchers may utilize an intermediary fluorescent compound; this intermediary’s absorption spectrum should overlap with the CL compound emission and its emission spectra better overlaps the PS absorption, thereby enacting a second RET step (Watrob et al., 2003b). While additional energy is lost as a result of a second RET step, it is possible that the kinetics from the increased overlap in absorption/emission spectra between the CL compound/intermediate and intermediate/PS as compared to the CL compound/PS overlap contribute to enhanced CL and as a result enhanced PS activation (Watrob et al., 2003b). Such couplings have also been shown to have greater sensitivity and efficiencies in biological systems (Hu et al., 2015). Moreover, the presence of an intermediate does not exclude direct CL/PS RET, but merely supplements for increased efficiency (Watrob et al., 2003b; Galperin et al., 2004; Chen et al., 2009; Hu et al., 2015).

**FIGURE 4:**
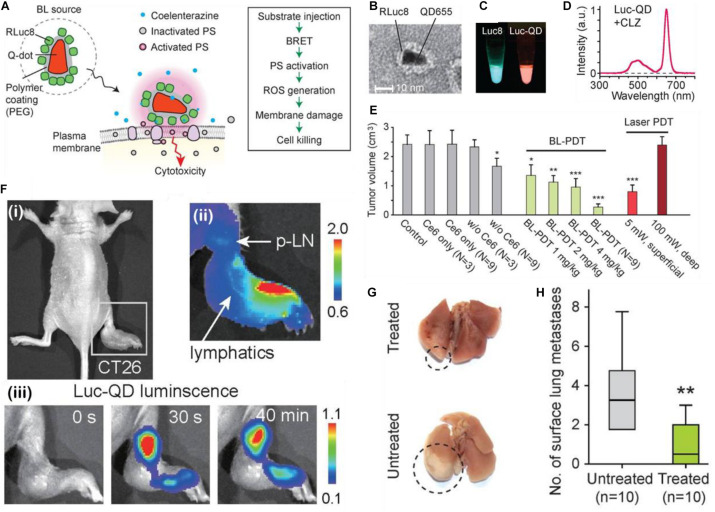
**(A)** Schematic of the design and cell killing action of Luc-QDs. **(B)** TEM image of Luc-QDs. **(C)** Chemiluminescent spectra of the Luc8 coelenterazine complex (left) and the post-FRET, redshifted Luc-QD (right) solutions and **(D)** associated red-shifted emission spectrum. **(E)** End point tumor volumes for controls, BL-PDT, and laser PDT. **(F)** (i) Footpad CT26 tumor model of a mouse before injection; (ii) Luminescence images immediately after injection at of the Luc-QD complex as the distal part of the footpad tumor. Arrows showing the travel *via* lymphatic system to the proximal lymph node; (iii) Luc-QD fluorescence traveling to and remaining in the lymph node up to 40 min. **(G)** Images of Luc-QD treated and untreated LN LCC-GFP tumor bearing mouse lungs, with **(H)** associated quantification of lung metastases (Kim et al., 2015). *p < 0.05, **p < 0.01, ***p < 0.001.

As might be expected, quantum dots are a very appropriate choice for playing the part of a FRET intermediate. Their small size, customizable absorption/emission bands allow for a broader utilization of photosensitizers which may be activated. In this work done by Kim et al. (2015) a chemiluminator RLuc8 is conjugated to a quantum dot with a maximum absorption at 655 nm (Figures 4A,B), which shifted the effective emission peak from blue light (peak around 500 nm) to around 670 nm, permitting the Luc-QD complex to excite the Ce6 PS; since the redshifting of the chemifluorescence signal can be observed in the Luc8-QD combination (Figures 4C,D). The nanotherapeutic agent was then applied to a tumor mouse model; the *in vivo* tests show that by increasing the RLuc-QD dosage, and by increasing the number of subsequent treatments *via* RET PDT (*N* = 9), the resulting tumor suppression is more significant than three treatment (*N* = 3) of superficially illuminated PDT (Figure 4E); the single dose equivalent to the conventional PDT for single treatment is 4 mg/kg, an uncommonly high dose. These results seem to suggest that it requires at least three times the dosage of the Luc-QD as compared to a conventional PDT treatment. However, in this paper, the authors stress that this application is designed for deep-seated tumors, and in this treatment scenario, they used a sham of tissue 5 mm thick to stress that even a small addition of simulated depth renders conventional PDT ineffective. What is most interesting about this report is the alternative application of the Luc8-QD PDT system wherein the authors noticed, from chemiluminescent imaging, that the Luc8-QD nanoparticle system drained into the nearby lymph node after injection into a footpad tumor (Figure 4F) which contributed to lower mortality. This resulted in the reduction of lung metastases when the Luc-QD PDT system was applied (Figures 4G,H). This result is interesting in that it provides a plausible mechanism by which an overall mouse survival curve can be influenced by factors other than main tumor growth suppression. In this case, the researchers diligently observed a characteristic of the nanoparticle auto-PDT system and was able to induce a reasonable conclusion about the broad mechanism of increasing mouse survival.

While there is an increase in complexity with the inclusion of a secondary electronic excitation step, there are undeniable increases in chemiexcited PDT effect, but these gains would likely depend heavily on the type of system being considered (Sadhu et al., 2010; Hu et al., 2015). For example, if a donor and acceptor were chosen based on some other criterion other than emission-absorption overlap (e.g., biocompatibility or possessing some desired functional group) the inclusion of a FRET intermediary would allow for effective excitation of the acceptor from the donor. Two stage PS excitation has some advantages, but it is possible that smart choice of donor and PS, or their rational coupling considering orientation/distance with respect to each other would lead to higher efficiencies. Certainly, the maximum achievable theoretical efficiency ought to be higher with fewer energy transfers *via* a FRET-based mechanism.

### Cherenkov Radiation Energy Transfer (CRET)

Cherenkov radiation is a phenomenon associated with beta emission from radioactive particles that was first described and characterized by Cherenkov experimentally (Čerenkov, 1937). Cherenkov radiation arises when an emitted charged particle (usually an electron) moves through a dielectric medium (like water) at a speed faster than the speed of light in that medium (Spinelli and Boschi, 2015; Tanha et al., 2015). An electromagnetic field is always generated as a charged particle moves through a medium, and this can induce an associated polarization in the electrons of nearby atoms. This is not an excitation nor an ionization but the generation of spherical wavelets composed of *en masse* fluctuations of surrounding electrons from every point along the path of the original traveling charged particle (Jelley, 1955; Klein et al., 2019). When this particle exceeds the speed of light in the medium, these wavelets constructively interfere, not unlike a sonic boom, to produce electromagnetic waves in the visible wavelengths. The reason Cherenkov radiation is observed to be blue is because the intensity of this radiation is proportional to the frequency (Jelley, 1955). When this radiation is observed, it is of sufficient intensity to excite fluorophores, quantum dots, PSs, or any other molecule/material with an excitable bandgap in the blue light region (Bernhard et al., 2017; Shaffer et al., 2017).

Thus, Cherenkov radiation is also capable of acting as a donor in CRET. By utilizing radioactive isotopes with high beta-emissions (e.g., ^18^F, ^64^Cu, or ^68^Ga, ^90^Y, etc.), several research groups have been able to induce electronic excitation of fluorophores and photosensitizers. Much like FRET, CRET efficiencies are usually dependent on the concentration of the emitting isotope (the donor), the concentration of the fluorophore (acceptor), and the overlap between Cherenkov luminescence emission peak and the acceptor absorbance peak (Bernhard et al., 2017). However, the mechanism of Cherenkov radiation donating electronic energy to fluorophores is certainly not the same as chemiluminescent molecules donating to fluorophores given that the difference in the source excited electronic energy; one arises from an excited electronic state generated *via* chemical reaction, the other arising from the constructive interference of induced electromagnetic waves. Further, it is known that RET is a radiationless transfer of energy, and that Cherenkov arises from the propagation of radiation itself. Unfortunately, the exact mechanism of neither RET nor CRET are fully understood.

**FIGURE 5:**
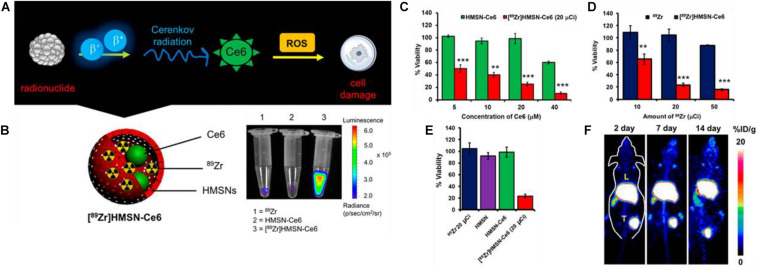
**(A)** Scheme showing the mechanism by which radionuclides in [^89^Zr]HMSN-Ce6 can induce ROS cell damage mediated by Cerenkov radiation excitement of PS Ce6. **(B)** Luminescence of [^89^Zr]HMSN-Ce6 vs. free [^89^Zr] and HMSN-Ce6 controls. **(C)** Cell viability after incubation with varying concentrations of [89Zr]HMSN-Ce6 and HMSN-Ce6 as controlled by Ce6 concentrations. **(D)** The same but controlling [^89^Zr] and [^89^Zr]HMSN-Ce6 concentrations. **(E)** Tumor growth suppression of [^89^Zr]HMSN-Ce6 and control treated mice. **(F)** Cerenkov radiation imaging of mice over a 14-day span post-injection (Kamkaew et al., 2016). **p < 0.01 and ***p < 0.001.

An example of using CRET to initiate PDT can be seen in the work by Kamkaew et al. (2016) wherein a mesoporous silica framework was loaded with beta radiation emitting ^89^Zr and the photosensitizer chlorin e6 (Ce6) for an auto-PDT nanosystem ([^89^Zr]HMSN-Ce6) as seen in Figure 5A. The loading of the radioisotope was achieved by surface chelation of the deprotonated silanol groups on the MSNs to the ^89^Zr. The [^89^Zr] loading was sufficiently high to excite the Ce6 PS (Figure 5B). Both Ce6 and [^89^Zr] loadings were directly were directly related to the cancer cell toxicity (Figures 5C,D) with increasing concentration of both lowering observed viabilities. However, the doubling concentration of ^89^Zr appeared to have a more dramatic effect between 10 and 20 μC, but little to no effect when doubling again; this contrasts with Ce6 loading which appears to show a monotonic decrease with each doubling up to 40 μM. The nanoparticle shell itself apparently imparting no toxicity on the cells (Figure 5E). When applied *in vivo*, significant PS fluorescence from both the tumor and liver can be observed, which shows that active CRET is occurring in these areas (Figure 5F); tumor growth was totally inhibited in the [^89^Zr]HMSN-Ce6 treatment group over a span of 14 days with tumors retaining their starting diameter. The disadvantages of this approach become evident from Figure 5F where active PDT is occurring both in the tumor and in the tumor tissue unlike CL based APDT.

What perhaps is most interesting about CRET-based APDT, is that despite the extremely low light fluence rate of Cherenkov luminescence related to β-radiation as compared to conventional PDT, significant cancer cell toxicity and tumor growth inhibition can still be observed (Gonzales et al., 2014; Glaser et al., 2015). It is not clear how markedly lower (several orders of magnitude) light intensities can still produce significant toxicities, but some authors are generally quick to point out that CRET based PDT is long lasting with isotope half-times commonly on the order of several days.

### External Radiation Applications

After examining the main processes, whereby the auto-excitation of the PS can occur *via* CRET or RET, it is worth mentioning a few modalities which are decidedly not APDT, as they require an external trigger to become activated and generate a therapeutic effect.

Afterglow PDT is generally described as set of compounds or nanomaterials which, after irradiation with an external light source, possess long-lived excited triplet states and can create singlet oxygen or other ROSs after irradiation has stopped. The types of materials used to form these afterglow PS generally consist of noble metalloorganic complexes, porphyrins, or carbon NPs, or most recently organic crystals (Fan et al., 2017; Liu et al., 2018; Wang et al., 2018; Xu et al., 2020; Yang J. et al., 2020). Zinc-Gallium-chromium nanoparticles are a popular modality, and have been shown to have phosphorescence half-lives of approximately 1 min (Fan et al., 2017). However, these materials still cannot be classified as APDT systems since they still require an initial excitation *via* an external lights source, eliminating the advantages offered by true APDT systems. In theory, if afterglow materials with long enough half-life are created, then these materials can be irradiated before injection into the body, effecting treating the irradiation step as a part of the preparatory synthetic procedure, and these materials could be considered APDT, however, the exponential decay of chemical excitation states of PSs ensures that they will always be most toxic upon administration into the blood stream (Ware et al., 1973), and will exhibit lesser toxicity as they accumulate in the desired, and sometimes undesired, regions.

X-ray is another technique which also requires external radiation in order to generate reactive oxygen species; this technique typically utilizes either a direct X-Ray to ROS generation (e.g., Cu-Cy metalloganic) or a scintillator intermediate which transfers the X-Ray radiation into infrared light which can be used with certain photosensitizers (SAO nanoparticles LaF materials, etc.) (Ma et al., 2014; Chen et al., 2015; Wang et al., 2016; Song et al., 2018; Sun et al., 2020). X-Ray based PDT also break the depth dependence, due to the low attenuation in tissue, but are still a form of externally applied radiation so these are not discussed further in this review.

## Formulation Scheme

The formulations are categorized by the quantity of steps within the APDT system of which there are three main parts: (1) the chemi- or radio-luminescent compound, (2) the optional inclusion of an intermediary RET compound, and (3) the photosensitizer itself. Many formulations choose to incorporate other catalysts or targeting functionalities which are not counted as directly relevant to APDT. Even the bioluminescent catalysts for luminol are not counted as a “component” of the APDT since luciferin itself undergoes the excitation.

### Single Component

In most papers, few utilize NPs for a singular purpose; these will be referred to as single component schemes. Single component nanoparticles systems for APDT, wherein only a single component of the APDT system is incorporated in the nanoparticle, can still exhibit high levels of apparent complexity. Take for example work done by Jiang et al. (2019) where they formulated a conjugated polymer poly[2-methoxy-5-(2ethylhexyloxy)-1,4-phenylenevinylene] (MEH-PPV) and co-precipitated it with poly(styrene-co-maleic anhydride) (PSMA) to produce nanoparticles. The nanoparticles were then modified with hemoglobin (Hb) using NHS/EDC coupling to create Hb-NPs. The Hb-NPs were then encapsulated in liposomes for administration. Luminol, the CL compound, was administered separately. In this scheme, while Hb is reported to catalyze the oxidation of luminol for APDT therapy, it is not a necessary component. Thus, a complex multi-component nanoparticle system actually possesses only one component of the APDT system, which is the photosensitizing polymer MEH-PPV in this case. Other components either merely improve cellular uptake or improve the reactive oxygen species conversion efficiency.

This paper and another by Kotagiri et al. (2015) show how the nanoparticle structure itself can also serve as a component of the APDT system. In this paper they utilize TiO_2_-Transferrin-Titanocene (TiO_2_-Tf-Tb) NPs for APDT excited by Cerenkov radiation originating from ^18^F or ^64^Cu atoms, with ^64^Cu giving enhanced PDT outcomes as compared to ^18^F. While the transferrin provided targeting capabilities, the titanocene, a failed clinical chemotherapeutic which was shown to create peroxyl radicals when excited by light radiation, provided a secondary route of ROS generation, with the primary originating from TiO_2_’s intrinsic ability to generate singlet oxygen when exposed to radiation; the TiO_2_-Tf-Tb NPs were able to generate impressive anti-cancer and anti-tumor effects.

Single component systems need not merely include the PS itself of course, and many choose to conjugate a bioluminescent catalyst to the nanoparticle. These systems can still be considered single-component APDT systems since the catalyst, while enabling chemical oxidation of the substrate toward chemical excitation, is not technically involved in the transfer or generation of ROS. Such are the cases for Yang Y. et al. (2018) and Al-Ani et al. (2019) where the biocatalyst is attached to a photosensitizing polymer nanoparticle and a protoporphyrin functionalized protein nanoparticle, respectively.

In the above examples for single component systems, the photosensitization often serves as the single component function of the nanoparticle. There are several examples of nanoparticle-based systems where the RET intermediate serves as the single-component nanoparticle, most obviously for the case of QD type nanoparticles. Such examples have been published in papers like those by Hsu et al. (2013) and Kim et al. (2015) as already discussed where the QDs are conjugated to luciferases to catalyze the luciferin excitation and thus transfer to the QD RET intermediate.

### Bi-Component

In terms of nanoparticle formulations, most seek to combine at least two aspects of the APDT system; these can be collectively referred to as coupled schemes, or bi-component schemes.

#### PS and Chemi-Exciting Compound

**FIGURE 6:**
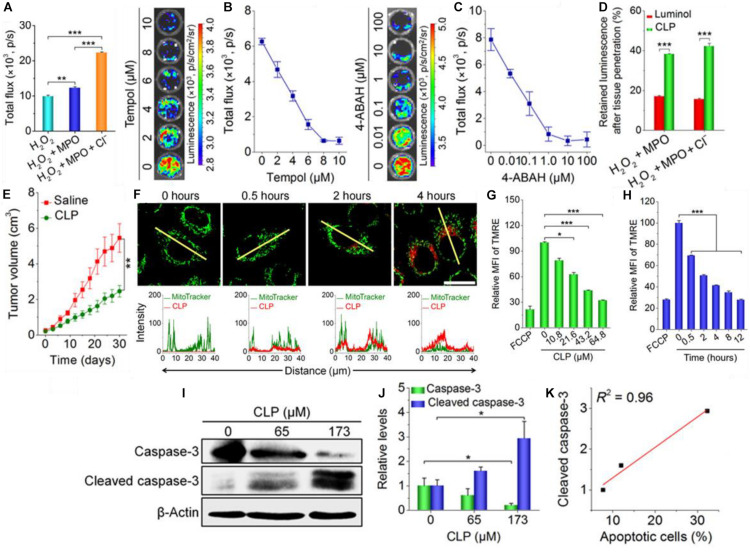
**(A)** The PEG-Ce6-Luminol conjugate and its self-assembly into a CLP nanoparticle for PDT. **(B)** The chemiluminescence of the CLP NP in the presence of hydrogen peroxide, H_2_O_2_ plus MPO, and H_2_O_2_ plus MPO + Cl^–^ and the measured chemiluminescent flux of light. **(C)** The inhibition of CLP chemiluminescence via 4-ABAH, an MPO inhibitor. **(D)** The inhibition of CLP chemiluminescence via Tempol, a ROS scavenger. **(E)** Tumor growth suppression of a A549 lung cancer xenograft tumor treated by CLP NPs vs. a saline control. **(F)** Confocal microscopy images (upper) of cells stained with MitroTracker Green after 0–4 h of incubation with CLP NPs with fluorescent quantification (lower) along the yellow line; CLP NPs show apparent colocalization with the tumor cell mitochondria. **(F)** The mean fluorescence intensity (MFI) of tetramethylrhodamine ethyl ester (TMRE), a mitochondrial membrane fluorescent probe, as a function of CLP NP concentration; carbonyl cyanide *p*-trifluoromethoxy phenylhydrazone (FCCP) serves as a positive control as it is a known mitochondrial membrane depolarizer. **(H)** The same as **(G)** but with increasing incubation time. **(G)** Florescenc intensity as measured by flow cytometry of a indicator of mitochondrial membrane potential (TMRE). **(I)** Gel picture and **(J)** quantification of Western blot bands showing the relative amounts of caspsase-3 and cleaved caspsase-3 in A549 cells with increasing CLP NP concentration after ***P < 0.001; ns, no significance. *P < 0.05, treatment for 24 h. **(K)** The correlation between the amount of cleaved caspsase-3 and percent of total cell population that became apoptotic (Xu et al., 2019).

The most direct way of potentially increasing the FRET efficiency is by directly coupling the donator and the photosensitizer. In an exemplary paper written by Xu et al. (2019), they synthetically combined a luminol molecule with a Ce6 PS and a PEG tail. This allowed the Ce6-Luminol-PEG conjugate (CLP) to self-assemble into micelles which could enact auto-PDT. The CLP conjugate showed increasing chemiluminescence in the presence of H_2_O_2_, myeloperoxidase (MPO) and a chlorine salt (Figure 6A). MPO is a common peroxidase found in primarily neutrophils and monocytes which catalyzes the formation of ROSs including HOCl for microbial killing (Aratani, 2018). Inhibition of chemifluorescence *via* addition of either Tempol, an ROS scavenger, or 4-aminobenzoic hydrazide (4-ABAH), and MPO inhibitor, confirms the importance of this enzyme in generating ROS for luminol chemifluorescence (Figures 6B,C). Cellular uptake was improved significantly when luminol was included in the CLP nanoparticle (Figure 6D). When this group utilized CLP-NPs for PDT tumor treatment, they found a modest tumor growth suppression effect (Figure 6E), but what made the paper exemplary in this case was the following elucidation of the tumor suppression mechanism. Xu and coworkers found that there was significant co-localization of the CLP to the mitochondria (Figure 6F); increasing CLP dosage and incubation dose showed a related decrease in mitochondrial membrane potential as measured by a tetramethylrhodamine ethyl ester (TMRE) fluorescent probe suggesting a disruption of mitochondrial function (Figures 6G,H). Western blot analysis of the lysed cells showed that there was a concomitant increase in the amount of cleaved caspase-3 with increases in administered CLP concentration (Figures 6I,J), and that this increase in cleaved caspase could be correlated to the increase in percentage of apoptotic cells (Figure 6K). The authors proposed that the apoptosis in A549 cancer cells was induced by incubation with CLP NPs; the NPs are endocytosed within the cell then undergo chemiluminescence initiated by intracellular ROSs already present. Then, RET/BRET occurs and the PS Ce6 generates singlet oxygen which destabilizes the potential of the nearby mitochondria. The decreased potential finally causes caspase activation and cleavage which is a major pathway cell signaling apoptosis. This group published a follow up paper showing equally impressive results (An et al., 2020).

**FIGURE 7:**
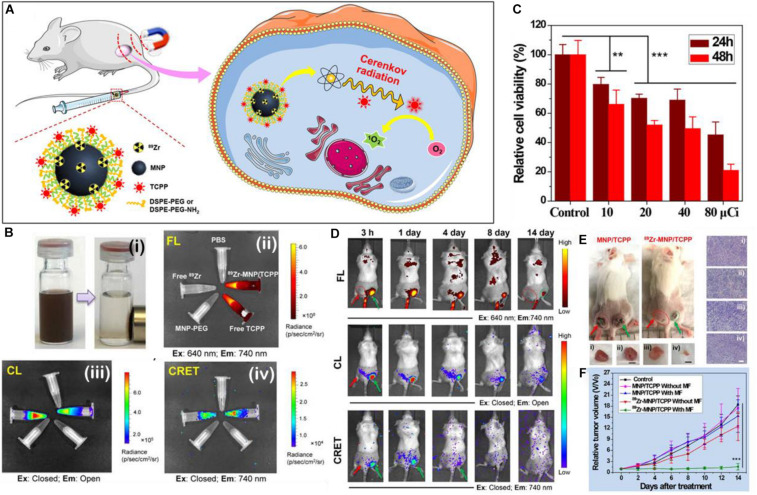
**(A)** A schematic illustration of the make-up, delivery, and the generation of singlet oxygen by ^89^Zr-MNP/TCPP. **(B)** Figure showing (i) a solution of ^89^Zr-MNP/TCPP with and without a magnet applied; (ii) the fluorescence of various solutions, clockwise: PBS control, ^89^Zr-MNP/TCPP, free TCPP control, MNP-PEG (no PS) control, and free ^89^Zr with TCPP as control; (iii) the Cherenkov luminescence (all wavelengths) of the same; (iv) CRET (740 nm emission only) of the same. **(C)** Cell viability after 24 and 48 h incubation with ^89^Zr-MNP/TCPP dose controlled by effective radiation dose. **(D)** Luminescent imaging of bilateral tumor bearing mice at various time points after injection with the ^89^Zr-MNP/TCPP as measured by fluorescent imaging (top), chemiluminescent imaging (middle), and CRET imaging (bottom). **(E)** Scheme showing a bilateral 4T1 tumor model of a BALB/c mouse: one tumor with applied external magnetic field (right, green arrow), and one without (left, red arrow). **(F)** associated tumor growth profiles (Ni et al., 2018). **p < 0.01 and ***p < 0.001.

PS and donor coupling Cherenkov platforms are some of the simplest auto-PDT platforms since no substrate or chemical is required for initiation of luminescence. As of yet, no Cherenkov radiation platforms have been published using a RET intermediate for PDT, but this can be done (Bernhard et al., 2017). Thus, all that is required is the radioactive isotope and the PS to be contained in the same platform. Ni et al. (2018) took the design a step further and created magnetic Zn-Mn-Fe_2_O_4_ nanoparticles with surface labeled [^89^Zr] as the beta radiation emitter and TCPP functionalized to the surface *via* a DSPE-PEG moiety (^89^Zr-MNP/TCPP) (Figure 7A). The ^89^Zr-MNP/TCPP retained significant magnetism in solution and the complex showed detectable RET *in vitro* (Figure 7B) as measured by the fluorescence of the TCPP PS in the absence of externally applied light. *In vitro* results confirmed that toxicity was related to the beta radiation intensity (Figure 7C). *In vivo* results of the magnetic induced targeting were impressive with a clear increase of the measured RET in a bilateral tumor model wherein the tumor to which a magnet was applied showed significant increase in the amount of RET and thus tumor growth suppression (Figures 7D,E). It is interesting that in the tumor growth suppression curves (Figure 7F), the tumors to which magnetism was not applied showed little suppression; this is in stark contrast to results published in other papers where targeting is not necessary for tumor growth suppression. This is probably due to insufficient CRET based PDT occurring in the tumor, but whether this is due to insufficient ^89^Zr-MNP/TCPP accumulation or insufficient PDT initiated events per ^89^Zr-MNP/TCPP complex is still debatable. Perhaps significant accumulation is required for Cherenkov radiation driven PDT outside what can be achieved without targeting or otherwise enhanced uptake.

Yu et al. (2018) have also utilized hollow mesoporous silica nanoparticles for the loading of the CPPO in perfluorocarbon along with the Ce6 PS concomitantly with glucose oxidase which generates hydrogen peroxide from the conversion of glucose to gluconic acid. Lin et al. (2019) claim to use fluorescein as a bioluminescent molecule for the excitation of a PCN-224 type metal organic framework NP, but as is commonly known, fluorescein does not luminesce without the application of external light. They nevertheless published results which show significant generation of cancer cell cytotoxicity and generation of ROS *in vitro* without light application. It is unclear whether this paper suffers from improper terminology usage, improper handling procedures which expose fluorescein to light, or is subject to other phenomena which generate ROS and cancer cell death. Berwin Singh et al. (2017) also published a formulation which encases peroxalate polymer and protoporphyrin in a F-127 amphiphilic polymer shell. The peroxalate polymer generates the dioxetanedione intermediary which directly excites the protoporphyrin that generates ROSs resulting in *in vitro* cancer cell death.

Another apparent rational coupling can be made between a CL compound with a PS in the presence of a bioluminescent catalyst; interestingly, there has only been one published example using this type of co-functionalization as done by Zhao et al. (2013a) In their work, by loading the luciferin, luciferase, photosensitizer all in a polymer microcapsule, they show a reduction in measured cancer cell toxicity as compared to administrating *via* a free luciferin solution. This is an interesting result; the free luciferin solution alone generated more toxicity than the microcapsule containing luciferin in equal concentration. This raises question about studies using externally administered luciferin in combination with a nanoparticle; cell toxicity experiments in other papers utilizing a luciferin-luciferase BRET excitation of PS may well have their results confounded by the toxicity of free luciferase. Control experiments where luciferin is omitted are common in these papers, but none show free luciferin by itself. Given the observed highly toxic effect it can have on cells, papers in this line of research ought to include luciferin control experiments.

#### RET Intermediate and PS

Lastly, the FRET intermediate and the photosensitizer can be coupled, as was done in work by Yang K. et al. (2019), wherein the photosensitizer PpIX was coupled to upconverting carbon nanodots (CDs). The emission of the fLuc, normally centered at 560 nm under basic conditions yielded significantly redshifted emission spectra (620 nm), and thus the wavelength was too low to excite the PpIX PS (abs. ∼380 nm). Thus, the upconverting CDs were functionalized to the PS *via* NHS/EDC coupling reaction. FRET transfer from the fLuc-luciferin to the CD is then upconverted to a wavelength of approx. 440 nm before a second RET transfer to the PpIX PS. The generation of ROS *via* PDT was measured with DPBF and showed significant generation of ROS during confocal microscopy. In a following paper by the same researcher, Yang K. et al. (2020) similarly used a CD-PS conjugation but with the PS being Ce6 instead, and also showed extensive emission and absorption characterization. The group again showed extensive cellular experiments, achieving 92% toxicity of SMMC-7721 cancer cells, but with no elucidation of the mechanism of cell death, assuming the mechanism was similar to that of H_2_O_2_ induced cell death. Here, the group expanded their work to include *in vivo* models showing that their PDT system decreased proliferating cell nuclear antigen and degree of vascularization which limited tumor growth to a size increase of approximately 40% as compared to 300–400% in control tumors. No explanation is given as to why these markers decrease with APDT treatment.

In a report by Zhang et al. (2014), they coprecipitated meta-tetra(hydroxyphenyl)-chlorin (m-THPC), a PS, an amphiphilic dendrimer, and semi-conducting polymer poly[2-methoxy-5-((2-ethylhexyl)oxy)-p-phenylenevinylene] (MEH-PPV) in order to create hydroxyl terminated photosensitizer doped polymer dots (HO-Pdots), which were then covalently linked to folic acid and horseradish peroxidase (FH-Pdots). The importance of this study lies in the specificity exhibited by preferential uptake and ROS generation of FH-Pdots in MCF-7 and C6 cancer cells as opposed to none in healthy normal NIH 3T3 cells; this is an important analysis omitted by many papers. While showing overall cancer cell toxicity is an acceptable step in showing therapeutic efficacy, many studies also only use cancer cells as measures for non-toxicity of controls. The real value in any therapeutic system, if it is to outcompete traditional chemotherapy, is showing minimal toxicity to healthy cells. This is suggested to become standard practice in future works. There is a conflict shown in this paper when compared to a similar work; Jiang et al. (2019) utilize MEH-PPV as the only method by which PDT can occur in their system, but using the same polymer. Zhang et al. (2014) show that this same polymer is not sufficient to sustain significant ROS generation. In fact, Zhang et al. (2014) claim that MEH-PPV is primarily responsible for the observable fluorescence signature emitted by their FH-Pdots, and in this system can function as a FRET intermediate, and is not responsible for the PDT effect. While it is true that any polymer or molecule with a band gap can undergo either fluorescence or intersystem crossing, many dedicated PDT systems nearly always utilize a PS that prefers the singlet oxygen generation route. Nevertheless, Jiang et al. (2019) still shows impressive cell toxicities toward HeLa cancer cells. Due to the differences in cell choice and ROS generation characterization techniques, direct comparisons cannot be made.

#### Tri-Component

Some authors choose to combine more than two aspects of the PDT scheme in tri-component schemes. Multicomponent schemes are relatively rare in the literature, likely due to their synthetic complexity and the sometimes unneeded inclusion of a RET intermediate; this increased complexity does necessarily create increased anti-cancer effect.

**FIGURE 8:**
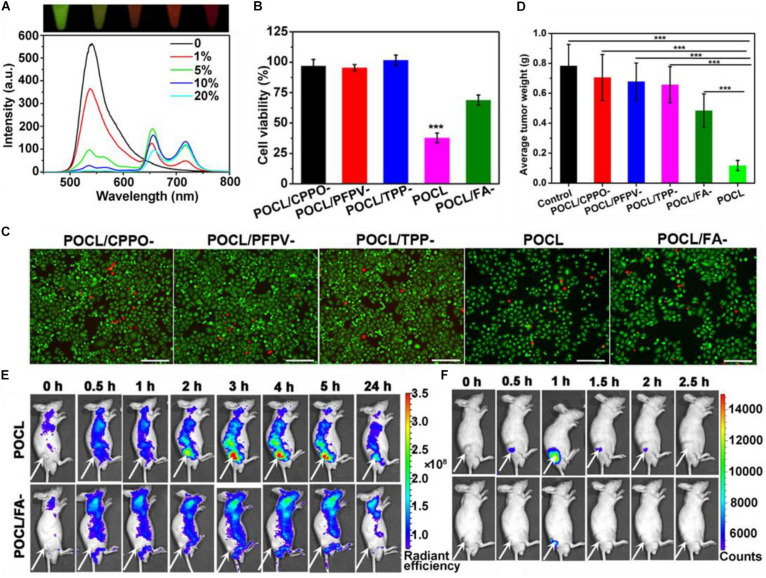
**(A)** The change in fluorescence emission spectrum of a POCL solution containing different TPP:PFPV ratios. **(B)** HeLa cancer cell toxicity of the POCL *sans* varying components as controls. **(C)** live/dead stain of HeLa cells. **(D)** End-point tumor size values for different treatments. **(E)** Fluorescence imaging of POCL or POCL/FA^–^ over time as compared to **(F)** chemiluminescent images (Wu et al., 2019). ***P < 0.001.

A notable example was published by Wu et al. (2019), where CPPO was used to directly excite poly[(9,9’-dioctyl-2,7-divinylene-fluorenylene)-alt-2-methoxy- 5-(2-ethyl-hexyloxy)-1,4-phenylene] (PFPV) *via* a CIEEL-like mechanism. PFPV then transfers the electronic energy *via* RET to the PS TPP, which, in the PDT productive pathway, undergoes intersystem crossing and electron transfer to generate singlet oxygen for cellular damage and death. As the amount of TPP is increased, the chemiluminescence of PFPV decreases as more energy is transferred *via* FRET to the PS (Figure 8A). The increased PDT efficiency can be clearly seen in Figure 8B where the absence of PFPV show similar cell toxicities as formulations without CPPO or TPP, with viabilities near 100%. This is because CPPO cannot chemically excite TPP. However, when PFPV is included to make the nanoparticle formulation (POCL), and even more so with folic acid (FA) attached, cell viabilities plummet as can be seen in live/dead assay microscopy (Figure 8C). Results here suggest that efficient cytotoxicity via PDT occurs primarily when the formulation is uptaken by the cell. *In vivo* results showed good tumor growth suppression as compared to relevant controls (Figure 8D). Better controls might include administration of a mixture of CPPO/PFPV/TPP without encapsulation in a nanoparticle capsule, but the authors were constrained by solubility limitations. In any case, the fluorescence signal of the PS TPP can be seen distributed throughout the body (Figure 8E), there is apparent targeting as far as the chemiluminescence of the APDT nanocomplex is concerned, again likely due to the oxidative conditions of the intratumor region (Figure 8F). This apparently sidesteps the issue brought up by Magalhães et al. (2016) wherein they claim that the use of APDT systems cannot be specific and thus do not have the advantages of conventional PDT.

## Discussion

The therapeutic efficacy and design of these APDT platforms are interesting, and have potential for further development in certain cases. APDT, or perhaps one might say nanoparticle theranostics in general, have recently been met with suspicion in terms of ability to translate successfully to clinic. In this respect, we feel that the potential of these APDT system lies in their ability to enhance diagnostic or therapy in a way beyond mere cancer treatment and tumor growth suppression. This potential can only manifest if researchers are diligent about understanding the underlying mechanism(s) by which APDT provides a therapeutic effect. The example set by Xu et al. (2019) where, despite seeing relatively modest tumor growth suppression when compared to other articles, they provide a plausible caspase cleavage mechanism by which tumor growth suppression is observed. The advantage of this is that now it is understood how the nanoplatform drives toxicity in this cancer models, allow for the derivation of formulation-therapy effects. This mechanism may be similar to toxicity induced by other types of nanoparticle formulations, or it may be different. Unfortunately, because many researchers choose to end their investigation with simply observing the differences in survival or tumor growth, comparison via nanoparticle formulation and cancer cell therapy is difficult; some of these measures concerning murine survival or tumor growth have been found to not correlate well with actual observed clinical success. Mechanistic characterization will allow researchers to derive structure-function-therapy relationships between nanoparticles and effects on the cellular environment.

In some cases of CL based excitation of PS, the unwillingness of some authors to verify the mechanism of excitation for their APDT system is problematic. While a full proof is not necessary, supporting evidence for the method of excitation of the CL and the following transfer to the PS should be included in all papers. Besides this, it is frequently difficult to discern what plays the role of the substrate, luminescence source, the transfer agent, if present, and the acceptor/ROS generator in some papers. While some papers clearly include a proposed mechanism or scheme of the proposed method of therapy, some do not and some are quite ambiguous about which aspect of their own, often quite complex, nanoparticle system fulfills which requirements for effective APDT. On a related note, the authors did not find in their search any current reports directly coupling CL compounds and a RET intermediate in a nanoparticle formulation, so this section is omitted in this report. This is curious given the apparent functional relation between these two components.

What is clear, broadly, at least, is that simple hydroxyl radical, singlet oxygen, and ROSs in general can cause cell toxicity in a number of ways (Huang, 2005; Da̧browski and Arnaut, 2015; van Straten et al., 2017). Does the structure, functionality, or method of excitation change the mechanism of cancer cell toxicity? Many of these questions are unclear, however, there has been at least one function that appears consistent: cellular uptake of APDT platforms is a critical component of their toxicity to cell *in vitro* and *in vivo*. As shown clearly in work by Wu et al. (2019), the FA moiety promotes cellular uptake and is used commonly as a functional group for the nanoparticle APDT system in many other papers. This is most probably because the generation of singlet oxygen, a short-lived molecular specie, or any other ROS is most effective when generated from within the cell, as it can more directly disrupt cellular processes from within.

One area where the literature is lacking greatly is a plausible explanation, or set of plausible explanations which account for the large discrepancy between expected light fluence in a tissue and the resulting PDT therapeutic effects. Both RET and CRET based treatments exhibit much lower (at least 100–1000x weaker) light fluence rates, below what is considered actionable for effective PDT (Schipper et al., 2006), yet nanoparticle based papers are still observing significant anti-cancer effects with supporting mechanistic proof of PDT-type disruption. It is entirely possible that the presence of the nanoparticle itself contributes to this toxicity or else modifies the excitation of the PS beyond what is considered typical in a theoretical model. Of course, biological and nanoscale systems are areas where the assumptions put forward by Forster for RET breakdown considerably (Scholes, 2003; Watrob et al., 2003a), which of course would alter the efficiency of PDT. Also, Cerenkov radiation is not generally subject to absorption in the same way as traditional light since the medium itself is what gives rise to the observed radiation. Future fundamental research ought to elucidate the precise mechanism by which these low fluence rates can give rise to such efficacious PDT, since hitherto the literature consensus is that the total energy transferred to the PS is almost certainly less than competing illuminated PDT. However, little seems to be known what the true “effective” dose appears to be and certainly even less is known how formulation within a nanoparticle system may affect this “minimum” excitation dosage. Given the repeated success in this field for anti-cancer therapy, it appears that pure illuminating power may not be the most critical factor in determining a PS’s ability to induce cytotoxicity. However, the ultimate goal of APDT is to remove the depth penetration issue of external illumination, so some researchers consider this point moot and so many of the proposed uses covered in this review are with regard to deep-seated or metastatic tumor models.

The advantages to using CRET for PDT and imaging lie in that no stimulus needs to be provided whatsoever to the luminescent compound, the beta-radiation emitting isotope, which functions totally independently of the microenvironment. Further, the RET-driven PDT is long-lasting for as long as the PS remain active and the particles remain in the body. The disadvantage is lack of specificity. As just mentioned, RET driven PDT occurs regardless of the environment. Since most CLs are able to generate excited electronic states in oxidative, or ROS-rich, environments, like those found in the tumor cell, a specific PDT treatment can still be accomplished, which is one of the main advantages of utilizing PDT in the first place. This can counter a main detraction of APDT in RET systems which says that APDT driven systems lose specificity (Magalhães et al., 2016). However, because the conditions inside tumor cells are considerably more oxidative than the rest of the body, perhaps this is not a limitation as far as some instances of RET-based APDT is concerned. For example, CPPO and luminol both generate excited electronic states from oxidation by ROSs, which are naturally elevated in some tumor tissues. Thus, oxidative intratumoral environments generate more excited electronic states which transfer *via* RET to the PS generating singlet oxygen for therapy. Wu et al. (2019) in the paper by Bin Liu and coworkers, there is a clear preference for the chemiexcitation of CPPO to occur only in the tumor microenvironment. This specificity is still retained as far as chemiluminescence generation of ROS.

Additionally, it is sometimes unclear how functionalizing PSs into nanoparticle system affects their ability to generate ROSs. While papers show generally an increase in intracellular ROS when using nanoparticle systems, this could result from increased intracellular uptake (phagocytosis) of nanoparticle complexes as compared with free PS; some papers omit these comparisons altogether, which is unacceptable as a formulation obviously needs to improve upon existing therapy in some concrete way. Further experiments should always use appropriate comparative controls showing how nanoparticles can induce a specific increase in efficacy of APDT. Further, the stability of the auto-PDT nanoparticle platforms is also problematic. While inorganic or inorganic components of NPs are unlikely to undergo significant degradation in the presence of ROSs or singlet oxygen, organic NP components are likely to do so. Little work has been done in this vein, and whether or not NPs are capable of resisting or undergoing breakdown in the biological environment is a major concern for translating nanomedicine to the clinic. Some CL and RET based systems show specific activity or therapy in tumor regions, but such action could also occur after partial or complete breakdown of the nanoparticle system *in vivo*.

As a final point of consideration, many papers seek to validate their methods of therapeutic efficacy by showing increased toxicity to cancer cells *in vitro* or *in vivo*. However, there is a second aspect to cancer nanotheranostics, which appears to be downplayed in favor of greater toxicity: PDT or any other therapy can induce toxicity in healthy cells as well, so a formulation that does not immediately generate greater *in vitro* toxicity should not necessarily be looked upon as a failure, as Zhao et al. showed that by modifying the amount and type of PS and the microcapsule environment, lower cell toxicity can be observed (Zhao et al., 2013a). Further, lowered toxicity may have resulted from the relatively poor uptake of the microcapsules into the cells, but the results are interestingly at odds with the rest of the literature. The other multi-component systems relevant to this review have already been discussed in previous sections.

Concluding, there is much research in the area of using Cherenkov radiation and chemiluminescent compounds for driving PDT in order to solve the depth penetration issue suffered by more conventional PDT methods. Nanoparticles can be beneficial for increasing the effectiveness of APDT by increasing the cellular uptake of PSs, increasing the tumor intracellular toxicity, and bringing together, *via* co-functionalization, the various components of a sometimes quite complex APDT platform. Success has been shown in several papers for the suppression of tumor growth in murine models despite the presence of conflicting results in an emerging field. Perhaps the root of these contradictions will be made apparent by characterizing the methods by which ROS generation by NP functionalized PSs can lead to cancer cell toxicity, as some groups have already started doing. Real promise in this area largely remains in the characterization of cancer cell specific toxicity, as compared directly to toxicity induced in normal healthy cells, and derivation of relationships between how nanoparticle formulation can influence the mechanism of induced toxicity in cells.

## Author Contributions

NB reviewed most of the literatures and wrote the majority of the main text. YZ provided several ideas about organization and topics, and contributed to parts of the text. JL and JQ discussed and edited the text. JL and PH provided the review direction, contributed original ideas for content, reviewed areas of the literature, and contributed to parts of the text. All authors contributed to the article and approved the submitted version.

## Conflict of Interest

The authors declare that the research was conducted in the absence of any commercial or financial relationships that could be construed as a potential conflict of interest.
